# Changes in ADF/destrin expression in the development of hair cells following Atoh1-induced ectopic regeneration

**DOI:** 10.3892/etm.2013.1089

**Published:** 2013-04-29

**Authors:** KAI JIN, DONG-DONG REN, FANG-LU CHI, JUAN-MEI YANG, YI-BO HUANG, WEN LI

**Affiliations:** 1 Department of Otolaryngology, Otology and Skull Base Surgery;; 2 Department of Lab Centre, Eye, Ear, Nose and Throat Hospital, Fudan University, Shanghai 200031, P.R. China

**Keywords:** actin depolymerizing factor/destrin, hair cell, kinocilium, planar cell polarity

## Abstract

The aim of this study was to investigate the effects of actin depolymerizing factor (ADF)/destrin and position changes of kinetosomes in the development of hair cells following Atoh1-induced ectopic regeneration in the basilar membrane of mice. We observed through immunofluorescence at various time-points the expression of ADF/destrin and the specific kinetosome marker, γ-tubulin, in hair cells following ectopic regeneration induced by adenovirus transfection, overexpression of Atoh1 and *in vitro* culture. Changes of ADF/destrin distribution and kinetosome position during *in vitro* culture of new hair cells [Myo7a(+)] following Atoh1-induced ectopic regeneration are consistent with the changes in ADF/destrin expression and the polar migration of kinetosomes in hair cells of the cochlear sensory epithelium in normal development. ADF/destrin is involved in the development of the auditory epithelium and the development and structural rearrangement of ectopically regenerated hair cells in mammals. The kinetosomes of hair cells following Atoh1-induced ectopic regeneration show positional changes *in vitro* at different time-points.

## Introduction

As one of the most frequently identified hearing disorders, sensorineural hearing loss is mainly caused by the loss of cochlear hair cells in the inner ear. In order to understand its pathology and develop therapies for hearing restoration, researchers have made great efforts to investigate different methods for the regeneration of hair cells using appropriate animal models, including the transdifferentiation of sustentacular cells to hair cells or the conversion of multipotential stem cells to hair cells (1,2). As the most frequently used and extremely maturely applied gene in the study of this field, Atoh1 is a basic helix-loop-helix (bHLH) transcription factor required for differentiation of hair cells in the inner ear. A previous study demonstrated that ectopic expression of Atoh1 at various time-points of tissue culture or *in vivo* culture of the cochlear basilar membrane or vestibular sensory epithelium induces additional regeneration of hair cells (3). Actin depolymerizing factor (ADF) plays an important role in numerous cell treatment processes requiring cytoskeletal rearrangement, including cell migration (4,5). A number of studies have investigated the function of ADF in morphogenetic events of organ polarity. Research findings of Kuure *et al* demonstrated that knockout of the ureteric bud (UB) epithelium cofilin1 gene (*Cfl1*) or inactivating mutations of the destrin gene (*Dstn*) has no effect on kidney morphogenesis; however, simultaneous deletion of the two genes interrupts the morphogenesis of branching structures in early development, and simultaneous deletion of UB epithelium *Cfl1* and *Dstn* (double knockout) leads to the accumulation of filamentous actin, damage of the normal epithelial structure and defects in cell migration (6). Twinstar (TSR) encodes *Drosophila* cofilin/ADF. The retina of the *Drosophila* TSR mutant is shorter compared with that of normal *Drosophila*, which is due to a lack of the stretching process required for retinal development. In the TSR mutant, the sensing rod structure is not disordered; however, it is wider than the normal structure. Adhesion connects photoreceptor cells with each other; however, the structure remains wider than that in the normal control group since the retinal stretching phenomenon is inhibited (7,8). However, at present, no study has been performed to investigate the expression of ADF/destrin in the development of hair cells following ectopic regeneration induced by the overexpression of Atoh1. Additionally it is also unknown whether the ciliary structure of these ectopically regenerated hair cells undergoes the planar cell polarity (PCP) process. The aim of this study was to not only analyze the position changes of ADF/destrin in hair cells following ectopic regeneration induced by overexpression of Atoh1, but also to examine whether the kinetosome position in these new hair cells [Myo7a(+)] following ectopic regeneration has polar migration, similar to that of hair cells in normal development. This should provide an important foundation for future in-depth investigation of the polarized growth of ciliary bundle structures and functional ion channels arising from ciliary bundle development in hair cells following ectopic regeneration.

## Materials and methods

### Sample collection, tissue culture and viral transfection

Male and female healthy closed colony C57BL/6 mice were provided by Shanghai SLAC Laboratory Animal Co., Ltd (Shanghai, China). This study was performed in strict accordance with the recommendations in the Guide for the Care and Use of Laboratory Animals of the National Institutes of Health. The animal use protocol was reviewed and approved by the Institutional Animal Care and Use Committee (IACUC) of Fudan University. The cochlear basilar membrane, utricle and ampulla *canalis semicircularis* were rapidly removed under the dissecting microscope. A sterilized coverslip coated with 0.1% polylysine (Sigma, St. Louis, MO, USA) was placed in a 35×10-mm culture dish (Falcon, Franklin Lakes, NJ, USA), to which 1.0 ml serum culture fluid was added and the basilar membrane was transferred in such a manner that it was adherent to the wall; then it was incubated at 37°C overnight. The following day, the serum culture fluid was replaced with serum-free medium [Dulbecco’s modified Eagle medium/nutrient mixture F-12 (1:1), supplemented with 20 ng/ml B27; Gibco, Carlsbad, CA, USA], to which the Ad5-enhanced green fluorescent protein (EGFP)-Math1 or Ad5-EGFP virus was transferred, so that the final concentration of virus (SinoGene, Beijing, China) was 1.0×10^8^ PFU. Then, the culture fluid was replaced once every 1–2 days depending on the growth of the tissue adherent cells, followed by incubation in a quiet environment at 37°C and 95% humidity with 5% CO_2_. On the sixth (DIV6) and twelth day (DIV12) after viral transfer, the culture dish was removed for fixed observation. Myo7a(+) cells (Atoh1-induced new ectopic hair cells) were obtained from the cochlear basilar membrane. During culture of neonatal cochlear basilar membrane *in vitro*, the basilar membrane culture was transfected with (EGFP)-Math1 or Ad5-EGFP to induce new Myo7a(+) cells in the culture *in vitro*.

### Immunofluorescent staining of cells

The cells were stained as follows: i) The medium was removed, the cells were washed once with phosphate-buffered saline (PBS) and immobilized with 1 ml 2% paraformaldehyde for 15 min. Then, the immobile liquid was removed, the cells were treated with 1 ml 0.2% Triton X-100 for 1 min, washed three times with PBS and sealed with 1% bovine serum albumin (BSA) for 1 h. ii) The primary antibody (monoclonal rabbit anti-ADF/destrin antibody; 1:200) was formulated with 5% donkey serum [prepared with 0.1% TritonX-100 and 1X PBS (PBST)], added to the cells and then placed in a refrigerator at 4°C overnight. iii) The cells were rinsed three times with PBST solution at room temperature for 2 h each time. iv) The secondary antibody (donkey anti-mouse Rho; 1:1,000) formulated with PBST was added to cells and then placed in a refrigerator at 4°C overnight. v) The cells were rinsed three times with PBST solution at room temperature for 2 h each time. vi) Fluorescein isothiocyanate (FITC)-conjugated phalloidin (l:500) formulated with PBST was added to cells which were then left at room temperature for 30 min. vii) The cells were rinsed with PBST solution at room temperature for 20 min; and viii) the tissue was transferred with fluoromount-G slide mounting medium and a small amount of Dow Corning high-vacuum grease was applied at the four corners. A coverslip was placed onto the slide and nail polish was applied around the edge.

### Whole-mount preparation and immunofluorescent staining of the tissue

The tissue was stained as follows: i) 10% donkey serum was formulated with PBST, added to the tissue and left at room temperature for 60 min. ii) The primary antibody (monoclonal rabbit anti-ADF/destrin antibody; 1:200) was formulated with 5% donkey serum (prepared with PBST), added to the tissue and then placed in a refrigerator at 4°C overnight. iii) The tissue was rinsed three times with PBST solution at room temperature for 2 h each time. iv) The secondary antibody [donkey anti-mouse Cy5 (1:1,000) and donkey anti-mouse rhodamine (1:1,000)] was formulated with PBST and added to the tissue which was then placed in a refrigerator at 4°C overnight. v) The tissue was rinsed three times with PBST solution at room temperature for 2 h each time. vi) FITC-conjugated phalloidin (l:500) formulated with PBST was added to tissue which was then left at room temperature for 30 min; vii) the tissue was rinsed with PBST solution at room temperature for 20 min. viii) The tissue was transferred with fluoromount-G slide mounting medium and a small amount of Dow Corning high-vacuum grease was applied at the four corners. A coverslip was placed onto the slide and nail polish was applied around the edges.

### Laser scanning confocal microscopy

**Figure 1.**
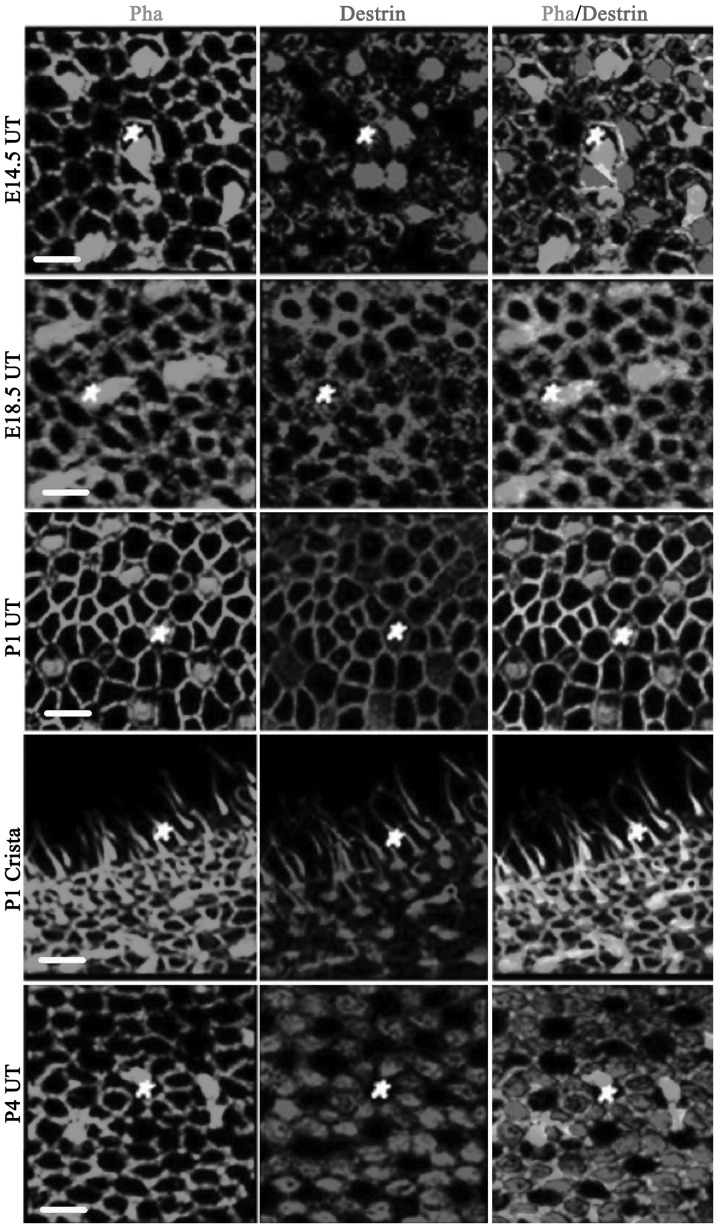
Expression level of ADF/destrin in the cochlear auditory epithelium of C57BL/6 mice from the embryonic period to various development stages after birth, as indicated by immunofluorescence. ADF, actin depolymerizing factor Pha, phalloidin; UT, utricle; En, nth day of embryonic development; Pn, nth day after birth. Bar, 10 *μ*m.

**Figure 2.**
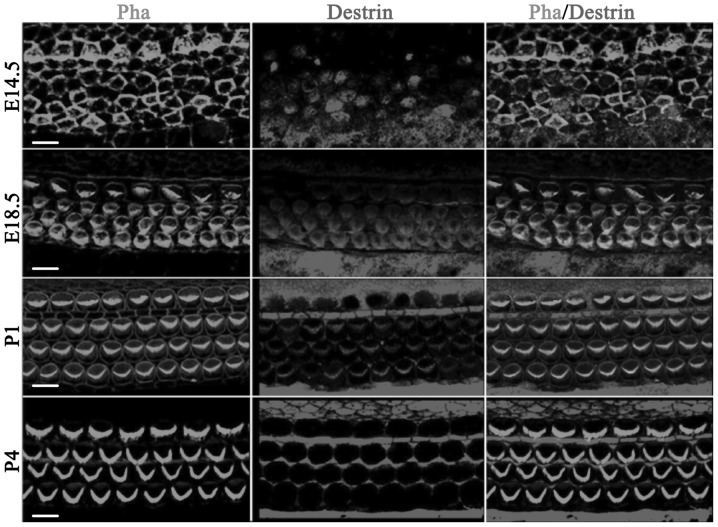
Expression level of ADF/destrin in the vestibular sensory epithelium of C57BL/6 mice from the embryonic period to various developmental stages after birth, as indicated by immunofluorescence. ADF, actin depolymerizing factor; Pha, phalloidin; En, nth day of embryonic development; Pn, nth day after birth. Bar, 10 *μ*m.

**Figure 3.**
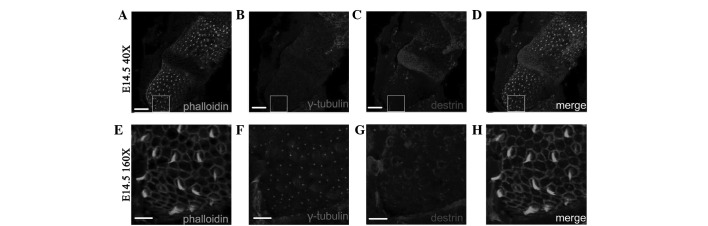
Expression and distribution of ADF/destrin and γ-tubulin in the ampulla *canalis semicircularis* of mice on day 14.5 of normal embryonic development (E14.5). ADF, actin depolymerizing factor. Bar (A–C), 50 *μ*m; bar (E–G), 10 *μ*m. The box indicates this area is magnified in the image below.

**Figure 4.**
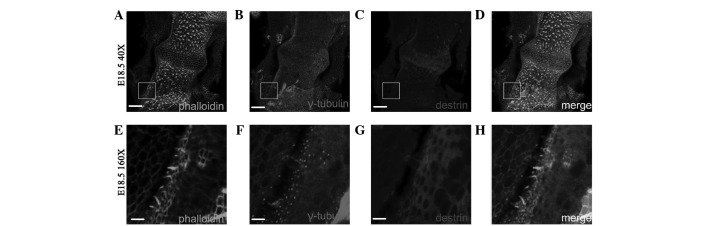
Expression and distribution of ADF/destrin and γ-tubulin in the ampulla *canalis semicircularis* of mice on day 18.5 of normal embryonic development (E18.5). ADF, actin depolymerizing factor. Bar (A–C), 50 *μ*m; bar (E–G), 10 *μ*m. The box indicates this area is magnified in the image below.

**Figure 5.**
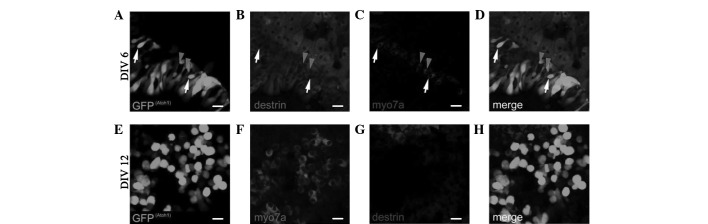
Expression change of ADF/destrin in Myo7a(+) cells following ectopic regeneration induced by overexpression of Atoh1 in the greater epithelial ridge of the cochlea. Gray arrowheads indicate destrin expression; white arrows indicate lack of destrin expression. ADF, actin depolymerizing factor. Bar, 10 *μ*m.

**Figure 6.**
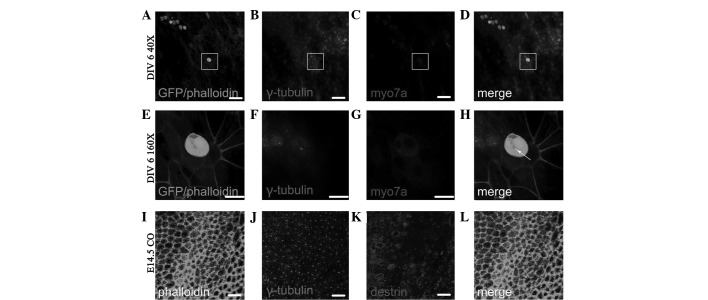
Kinetosome position of Myo7a(+) cells following regeneration induced by retroviral overexpression of Atoh1 and subsequent *in vitro* culture for 6 days, and kinetosome position of normal cochlear hair cells on day 14.5 of embryonic development. White arrow indicates γ-tubulin-labeled kinetosome located in the middle of an individual Myo7a(+) cell. Bar (A–C, I–K), 20 *μ*m; bar (E–G), 5 *μ*m. The box indicates this area is magnified in the image below.

**Figure 7.**
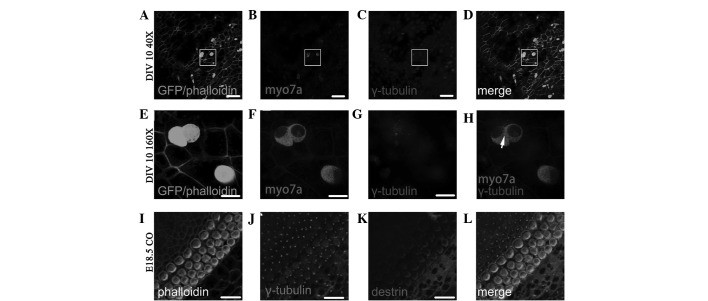
Kinetosome position of Myo7a(+) cells following regeneration induced by retroviral overexpression of Atoh1 and subsequent *in vitro* culture for 12 days, and kinetosome position of normal cochlear hair cells on day 18.5 of embryonic development. White arrow indicates γ-tubulin-labeled kinetosome located on the edge of an individual Myo7a(+) cell. Bar (A–C, I–K), 20 *μ*m; bar (E–G), 5 *μ*m. The box indicates this area is magnified in the image below.

The cochlear basilar membrane was scanned layer by layer from the uppermost layer down using a Zeiss LSM510 META laser scanning confocal microscope (Carl Zeiss, Oberkochen, Germany), with the laser at three wavelengths, specifically 488 nm (FITC), 543 nm (rhodamine) and 633 nm (Cy5), at a magnification of ×63, with the selected layer thickness at 0.5 *μ*m and an image resolution of 2,048×2,048. Figs. 1–7 were processed using LSM Image Browser (Carl Zeiss, Germany) and Adobe Photoshop 7.0.1 image processing software (Adobe, San Jose, CA, USA).

## Results

### Analysis of the expression of ADF/destrin in normal development of the cochlear basilar membrane in mice

Fig. 1 shows the distribution of ADF/destrin in the middle-bottom cochlea from day 14.5 of embryonic development (E14.5) to day 4 after birth (P4). On E14.5, destrin was scattered and expressed in sustentacular cells and hair cells, and on E18.5, destrin was expressed in cochlear hair cells, mainly distributed in the cuticular plate of hair cells and also expressed in a portion of the sustentacular cells. On P1, destrin was mainly expressed in the cilia of hair cells and inner phalangeal cells, and on P4, destrin was only expressed in the cytoplasm of sustentacular cells and no longer expressed in hair cells.

### Analysis of ADF/destrin expression in the normal development of vestibular utricles in mice

Fig. 2 shows the expression of ADF/destrin in the vestibule mainly comprising utricular macula epithelium, including *crista ampullaris* staining at P1. On E14.5, destrin was expressed in utricular epithelial hair cells and the surrounding sustentacular cells; however, it was mainly expressed in sustentacular cells. On E18.5, the cuticular plate of hair cells was stained and destrin was expressed in hair cells, sustentacular cells and the junction between sustentacular cells (co-localization of destrin and phalloidin-labeled actin was observed and the color became yellow). On P1, destrin was also significantly expressed in the cuticular plate of hair cells and the junction between the cells on the utricular macula. The kinocilium and cuticular plate were clearly stained on the *crista ampullaris*. On P4, destrin was significantly expressed in sustentacular cells (mainly in the cytoplasm). In Fig. 2, the white pentagons are hair cells.

### Analysis of the expression of ADF/destrin in normal development of the ampulla canalis semicircularis in mice

Fig. 3 shows that on E14.5, ADF/destrin was expressed in hair cells of the sensory epithelium of the ampulla *canalis semicircularis* and surrounding sustentacular cells. ADF/destrin was mainly located on the edge of the cuticular plate and was mainly expressed in sustentacular cells.

Fig. 4 shows that on E18.5, ADF/destrin was mainly expressed in sustentacular cells of the ampulla *canalis semi-circularis*. As shown in Fig. 4G–H, a cavernous fluorescent shadow was observed where hair cells were located.

### Changes of ADF/destrin expression in the development of Myo7a(+) cells following ectopic regeneration induced by overexpression of Atoh1 in the basilar membrane of neonatal mice

The expression of ADF/destrin in the greater epithelial ridge (GER) cell area following retroviral overexpression of Atoh1 and subsequent *in vitro* culture for 6 days (DIV6), as shown in Fig. 5A–D, indicates that destrin was also expressed in certain areas of Myo7a(+) cells; however, it was not expressed in other parts of Myo7a(+) cells. As shown in Fig. 5E–H of the expression of ADF/destrin in the GER cell area following retroviral overexpression of Atoh1 and subsequent *in vitro* culture for 12 days (DIV12), destrin was not expressed in any Myo7a(+) cells. No cell or area where Myo7a (fluorescence) and destrin (fluorescence) coexisted was observed (Fig. 5H).

### Polarity change of kinetosome position in the development of Myo7a(+) cells following ectopic regeneration induced by overexpression of Atoh1

Fig. 6A–D shows the Myo7a(+) cells following ectopic regeneration induced by retroviral overexpression of Atoh1 and subsequent *in vitro* culture for 6 days (DIV 6; fluorescence colocalized area in Fig. 6D). As shown in the enlarged images in Fig. 6E–H, which are four times the area of the white squares in Fig. 6A–D, the γ-tubulin-labeled kinetosome is located in the middle of individual Myo7a(+) cells (dot in Fig. 6H) and the Myo7a(+) cells therein have two nuclei. Fig. 6I–L shows the distribution of γ-tubulin and destrin of cochlear auditory epithelium on E14.5 in normal development. As observed in the GER side in the upper left side and lesser epithelial ridge (LER) side in the lower right side of Fig. 6I–L, the majority of kinetosomes in hair cells and sustentacular cells are located in the middle of the cuticular plate, and the minority are located around the cuticular plate (dots in Fig. 6J and L represent the location of the kinetosome.

Fig. 7A–D shows the Myo7a(+) cells following ectopic regeneration induced by retroviral overexpression of Atoh1 and subsequent *in vitro* culture for 12 days (DIV 12; fluorescence colocalized area in Fig. 7D). As observed in the enlarged images in Fig. 7E–H, which are four times the area of the white squares in Fig. 7A–D, the γ-tubulin-labeled kinetosome is located on the edge of individual Myo7a(+) cells (dot in Fig. 7H). Fig. 7I–L shows the distribution of γ-tubulin and destrin in the cochlear auditory epithelium on E18.5 in normal development. As observed in the GER side in the upper left side and LER side in the lower right side of Fig. 7I–L, all kinetosomes in hair cells were uniformly located on one side of the cuticular plate in order, i.e., where the projection of the tallest stereocilia is located (dots in Fig. 7J–L represent the location of the kinetosome.

## Discussion

This study aimed to investigate the effect of ADF and position change of kinetosomes in the development of *in vitro* cultured hair cells following ectopic regeneration. A previous study observed that ADF/destrin expression has temporal and spatial variation in the normal development of cochlear and vestibular sensory epithelium in mice (9), suggesting that ADF/destrin is involved in the development and maturation of hair cells in the auditory and vestibular sensory epithelium, as well as the ciliary bundle on the cuticular plate of sustentacular cells and hair cells in mammalians. However, no studies have determined destrin expression changes and position changes of kinetosomes in the development of hair cells following ectopic regeneration *in vitro*. By investigating the ectopic regeneration of hair cells induced by overexpression of Atoh1 in the cochlear basilar membrane of adenovirally transfected neonatal mice, we determined that ADF/destrin is involved in the development of hair cells and the ciliary bundle on their cuticular plate following ectopic regeneration, as well as in the structural integration of regenerated hair cells and surrounding sustentacular cells.

According to the experimental results, the transient expression of ADF/destrin in cochlear hair cells in the embryonic period and Myo7a(+) cells in early ectopic regeneration induced by overexpression of Atoh1 suggests that ADF/destrin plays a role in regulating the regeneration and circulation of cytoskeletal actin in the early development of cochlear hair cells. Moreover, the spatiotemporal distribution variation of ADF/destrin during *in vitro* culture of Myo7a(+) cells following ectopic regeneration is consistent with the phenomenon that destrin is expressed in hair cells of the cochlear auditory epithelium in the embryonic period (E14.5–E18.5) of normal development and not expressed after birth (P4). ADF/destrin is only expressed in sustentacular cells on day 4 after birth or later in normal mice and destrin is not expressed in Myo7a(+) cells following ectopic regeneration and subsequent *in vitro* culture for 12 days. Destrin is only expressed in the cells surrounding Myo7a(+) cells and ADF destrin expression tends to be expressed first in hair cells and then in sustentacular cells in the development of Myo7a(+) cells following Atoh1-induced ectopic regeneration, suggesting that destrin is involved in the structural development and structural integration of regenerated hair cells and surrounding sustentacular cells. Additionally, we identified that hair cells following ectopic regeneration induced by overexpression of Atoh1, move their kinetosomes at different culture times (specifically labelled by γ-tubulin) and γ-tubulin-labelled kinetosomes appear in the middle of individual Myo7a(+) cells following ectopic regeneration in early overexpression of Atoh1 (cultured for <1 week after transfection of the adenovirus). Furthermore, the kinetosome of ectopically regenerated individual Myo7a(+) cells after being cultured for 1 week, moves to the cell edge, which may be the edge of the immature cuticular plate (Figs. 6 and 7). This phenomenon is consistent with the phenomenon that the kinetosome of normally developing hair cells in the cochlear auditory epithelium moves to the cuticular plate side in development.

A previous study identified that in dividing cells, primary cilia may determine whether cells re-enter the cell cycle or remain static (10). The differential suggestion of cilia generation time may affect the opportunities for the cells to respond to extracellular signals, which affect the cell fate. Verdoni *et al* identified through studies on *Dstn* mutant mice that a number of genes related to the cell cycle are upregulated in this mutant, and mutant mice mitotic period may be affected (11–13). The results of the current study demonstrated that ADF is not expressed in ectopically regenerated Myo7a(+) cells in later transfection of Atoh1 (12 days after transfection); however, it is expressed in the cell body closely adjacent to ectopically regenerated Myo7a(+) cells. It is hypothesized that Myo7a(+) cells and adjacent cells may have been in different differentiation stages or have entered different cell cycles. In addition, previous studies of our experimental group revealed that the polar core protein Vangl2 and E-cadherin protein P120 in planar cells are involved in convergent extension movements in the development of auditory receptors and vestibular sensory epithelium, and the deficiency of polar proteins in planar cells and cell adhesion proteins seriously affect the establishment of a complete cytoskeleton in the auditory epithelium and vestibular sensory epithelium (14–16). Actin cytoskeleton remodeling plays a direct and specific role in the cell location information conversion process (17–21). The actin remodeling pathway is involved in the process of decoding extracellular signal gradient information to PCP (22–28). Blair *et al* emphasized the genetic correlation between TSR (ADF/cofilin analogs) and the PCP pathway, and inferred that actin remodeling is a key step in the PCP generation mechanism. The required mechanism for their redistribution remains unknown; however, actin remodeling is involved in the redistribution of core PCP proteins (7,8).

There is no evidence to suggest that the kinetosome of Myo7a(+) cells following ectopic regeneration in the late culture of this experiment is located at the final projection of the highest point of the cilia bundle; however, we determined that as the incubation time progresses, the kinetosomes of individual hair cells following ectopic regeneration move from the center of the cell to the edge of the cell. This provides a foundation for further in-depth investigation of the polarized development of ciliary bundle structure and functional ion channels arising from ciliary bundle development in hair cells following ectopic regeneration, since the kinetosome position change is closely related to the maturation of functional stereocilia in hair cells.

The theoretical significance of the experimental results includes: i) support of the conclusion that the overexpression of Atoh1 may induce the ectopic regeneration of immature hair cells with certain functions, and that regeneration and circulation activities of actin in which ADF is involved exist in the differentiation and maturation of these ectopically regenerated hair cells; ii) actin regeneration activity has spatiotemporal differences with the development of these ectopically regenerated hair cells. Structural changes of the cytoskeleton caused by the spatiotemporal differences in the regeneration activities of this actin are likely to be involved in polar migration of kinetosomes or the ciliary bundle on the cuticular plate of regenerated hair cells; and iii) contribution to the study of development and maturation of differentiated ciliary bundles in hair cells, polarity development of ciliary bundle in ectopically regenerated hair cells and its potential mechanism.
